# Supplementary material for: Resting-State Functional Brain Connectivity Best Predicts the Personality Dimension of Openness to Experience
Source: Personal Neurosci. 2018 Jul 5;1:e6. doi: 10.1017/pen.2018.8 (PMC6138449; doi:10.1017/pen.2018.8)
Supplement: Supplementary file 1 [file S2513988618000081sup001.pdf]

Supplementary Table 1. *List of HCP subjects included in the present study.*

100206	100307	100408	100610	101006	101309	101915	102311	102513	102614	102715	102816	103010	103111	103212
103414	103818	104012	104416	105014	105115	105216	105620	105923	106016	106319	106521	106824	107018	107321
107422	108020	108222	108323	108525	109325	110007	110411	110613	111009	111211	111312	111413	111514	111716
112112	112314	112516	112920	113215	113316	113619	113922	114217	114318	114419	114621	114823	115017	115320
115724	115825	116726	117021	117122	117324	117930	118023	118124	118225	118528	118730	118831	118932	119025
119126	119732	119833	120111	120212	120414	120515	120717	121416	121618	121921	122317	122620	122822	123824
123925	124422	124826	125222	125424	125525	126325	126426	126628	127226	127327	127630	127731	127832	127933
128026	128127	128632	128935	129028	129129	129331	129634	130013	130114	130316	130417	130619	130720	130821
130922	131217	131419	131722	131823	132017	132118	133019	133625	133827	133928	134021	134223	134324	134425
134627	134728	134829	135124	135225	135528	135629	135730	135932	136126	136227	136631	136833	137027	137128
137229	137532	137633	137936	138130	138231	138332	138534	138837	139233	139637	139839	140117	140420	141422
142828	143224	143325	143426	143830	144125	144226	144428	144832	144933	145127	145632	145834	146129	146331
146432	146533	146735	146836	146937	147636	147737	148032	148133	148335	148436	148840	148941	149236	149337
149539	149741	149842	150423	150625	150726	150928	151425	151627	151829	151930	152225	152427	152831	153025
153126	153227	153429	153631	153732	153934	154229	154431	154532	154734	154835	154936	155635	155938	156031
156334	156435	156536	157336	157437	157942	158035	158136	158338	158540	158843	159239	159340	159441	159744
159946	160123	160729	161630	161731	162026	162228	162329	162733	162935	163129	163432	164030	164636	164939
165032	165436	165638	165840	165941	166438	167036	167238	167440	167743	168139	168341	168745	168947	169343
169444	169545	169747	169949	170631	171330	171633	172029	172130	172332	172433	172534	172938	173334	173435
173536	173637	173738	173839	173940	174437	174841	175136	175237	175338	175540	175742	176037	176441	176542
176744	176845	177140	177241	177645	177746	178142	178243	178748	178849	178950	179245	179346	180230	180432
180533	180735	180836	180937	181131	181232	182032	182739	182840	183034	185038	185139	185341	185442	185846
185947	186040	186141	186444	186545	186848	187143	187345	187547	187850	188145	188347	188448	188549	189349
189450	189652	190031	191033	191235	191336	191841	191942	192035	192237	192439	192540	192641	192843	193239
193845	194140	194443	194645	194746	194847	195041	195445	195647	195849	195950	196144	196346	196750	196952
197550	198047	198350	198451	198653	198855	199251	199352	199453	199655	199958	200008	200109	200311	200513
200614	200917	201111	201414	201515	201818	202113	202719	202820	203418	203923	204016	204218	204319	204420
204521	204622	205119	205725	205826	206222	206323	206525	206727	206828	206929	208024	208125	208226	208327
208630	209127	209228	209329	209935	210011	210112	210617	211114	211215	211316	211417	211619	211821	211922
212015	212318	212419	212823	213017	213421	213522	214019	214221	214423	214524	214625	214726	217126	217429
219231	220721	221319	224022	227533	228434	231928	237334	238033	239136	239944	245333	246133	248339	249947
250932	251833	255639	255740	256540	257542	257845	257946	263436	268749	268850	270332	274542	275645	280739
280941	281135	283543	285345	285446	286347	286650	287248	289555	290136	293748	297655	298455	299154	299760
300618	300719	303119	303624	304020	304727	305830	307127	308129	309636	310621	314225	316633	316835	317332
318637	320826	321323	322224	325129	329440	329844	330324	333330	334635	336841	339847	341834	342129	346945
348545	349244	350330	352738	353740	356948	358144	360030	361234	361941	365343	366042	368551	368753	371843
376247	377451	378857	379657	380036	381038	381543	385046	385450	386250	387959	389357	391748	392750	393550
394956	395251	395756	397154	397760	401422	406432	406836	412528	413934	414229	415837	419239	421226	422632
424939	429040	432332	436239	436845	441939	445543	448347	449753	453441	454140	456346	459453	461743	463040
467351	469961	475855	479762	480141	481042	481951	485757	486759	497865	499566	500222	506234	510326	512835
513130	513736	516742	517239	518746	519647	519950	520228	522434	523032	524135	525541	529549	529953	530635
531536	531940	536647	540436	541640	541943	545345	547046	548250	552241	552544	553344	555651	555954	557857
558657	558960	559053	559457	561242	561444	561949	562446	565452	566454	567052	567759	567961	568963	570243
571144	572045	573249	573451	578057	579665	579867	580044	580347	580650	580751	581450	583858	585256	585862
586460	587664	588565	589567	590047	592455	594156	597869	598568	599065	599469	599671	601127	604537	611938
613538	615441	615744	616645	617748	618952	620434	622236	623844	626648	627852	628248	634748	635245	638049
644246	645450	645551	647858	654350	654552	654754	656253	656657	657659	660951	663755	664757	665254	667056
668361	671855	672756	673455	675661	677766	679568	679770	680452	683256	685058	687163	690152	692964	693764
694362	695768	700634	702133	704238	705341	707749	709551	715041	715647	715950	720337	723141	724446	725751
727553	727654	728454	729254	729557	731140	732243	734045	737960	742549	744553	748258	749058	749361	751348
751550	753150	753251	756055	757764	759869	761957	763557	765056	765864	767464	769064	770352	771354	773257
774663	782561	783462	784565	788674	789373	792564	792766	793465	800941	802844	803240	809252	812746	814548
814649	815247	816653	818455	818859	820745	825048	825553	825654	826353	826454	827052	828862	832651	833148
835657	837560	841349	843151	844961	845458	849264	849971	852455	856766	857263	859671	861456	865363	867468
870861	871762	871964	872158	872562	872764	873968	877168	877269	878776	878877	880157	882161	884064	885975
886674	887373	888678	889579	891667	894067	894673	894774	896778	896879	898176	899885	901139	901442	902242
904044	905147	907656	908860	910241	910443	911849	912447	917255	917558	923755	926862	927359	930449	932554
933253	942658	943862	951457	952863	955465	957974	958976	959574	962058	965367	966975	969476	970764	971160
978578	979984	983773	984472	987074	987983	989987	990366	991267	992673	992774	993675	994273	996782	

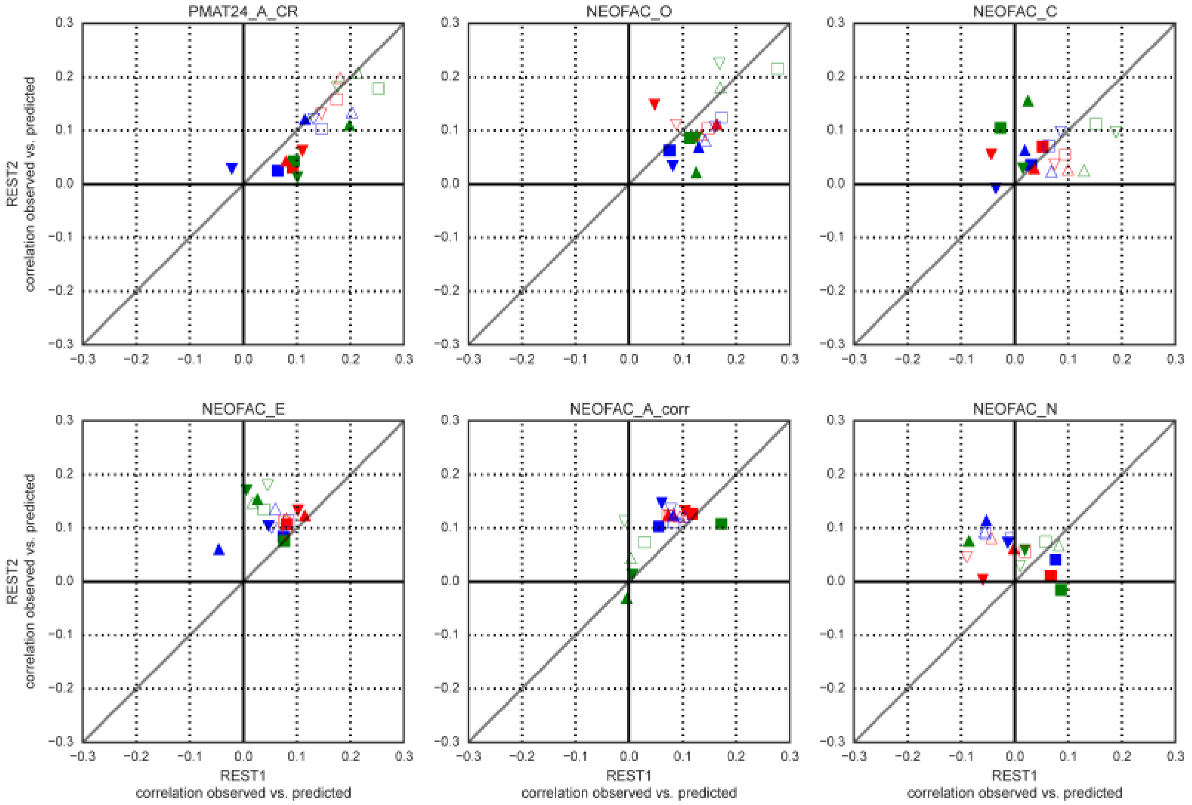

Color scale: −0.3 0 0.3

Parcellation	Model	Feature	PMAT24_A_CR REST1	PMAT24_A_CR REST2	NEOFAC_O REST1	NEOFAC_O REST2	NEOFAC_C REST1	NEOFAC_C REST2	NEOFAC_E REST1	NEOFAC_E REST2	NEOFAC_A_corr REST1	NEOFAC_A_corr REST2	NEOFAC_N REST1	NEOFAC_N REST2
MNI / Shen 2013	A	UNI +	0.09	0.03	0.08	0.06	0.05	0.07	0.08	0.11	0.12	0.13	0.07	0.01
		UNI -	0.06	0.03	0.08	0.06	0.03	0.04	0.07	0.08	0.06	0.10	0.08	0.04
		MULTI	0.09	0.04	0.11	0.09	-0.03	0.10	0.08	0.08	0.17	0.11	0.09	-0.02
	B	UNI +	0.08	0.04	0.16	0.11	0.04	0.03	0.11	0.12	0.07	0.13	-0.00	0.06
		UNI -	0.12	0.12	0.13	0.07	0.02	0.06	-0.05	0.06	0.08	0.12	-0.05	0.11
		MULTI	0.20	0.11	0.13	0.02	0.02	0.16	0.03	0.15	-0.00	-0.03	-0.09	0.08
	C	UNI +	0.11	0.06	0.05	0.15	-0.04	0.06	0.10	0.13	0.10	0.13	-0.06	0.00
		UNI -	-0.02	0.03	0.08	0.03	-0.03	-0.01	0.05	0.10	0.06	0.15	-0.01	0.07
		MULTI	0.10	0.01	0.13	0.09	0.01	0.03	0.01	0.17	0.01	0.01	0.02	0.06
MSM-All / Glasser 2016	A	UNI +	0.17	0.16	0.15	0.10	0.09	0.06	0.08	0.11	0.07	0.12	0.02	0.06
		UNI -	0.15	0.10	0.17	0.12	0.06	0.07	0.08	0.11	0.08	0.11	-0.05	0.09
		MULTI	0.25	0.18	0.28	0.22	0.15	0.11	0.04	0.13	0.03	0.07	0.06	0.07
	B	UNI +	0.18	0.20	0.13	0.09	0.10	0.03	0.08	0.12	0.10	0.12	-0.04	0.08
		UNI -	0.20	0.13	0.14	0.08	0.07	0.02	0.06	0.14	0.10	0.12	-0.06	0.09
		MULTI	0.21	0.21	0.17	0.18	0.13	0.03	0.02	0.15	0.00	0.04	0.08	0.07
	C	UNI +	0.15	0.13	0.09	0.11	0.07	0.04	0.08	0.09	0.08	0.12	-0.09	0.05
		UNI -	0.13	0.12	0.16	0.11	0.09	0.10	0.05	0.10	0.08	0.14	-0.01	0.08
		MULTI	0.18	0.18	0.17	0.23	0.19	0.10	0.05	0.18	-0.01	0.11	0.01	0.03

Supplementary Figure 1. Test-retest prediction results with minimal deconfounding. Only variables that are unlikely to be causally related to personality are regressed out of the scores: brain size, motion, and MB recon version.
